# Increasing of malignancy of breast cancer cells after cryopreservation: molecular detection and activation of angiogenesis after CAM-xenotransplantation

**DOI:** 10.1186/s12885-020-07227-z

**Published:** 2020-08-12

**Authors:** Xinxin Du, Plamen Todorov, Evgenia Isachenko, Gohar Rahimi, Peter Mallmann, Yuanguang Meng, Vladimir Isachenko

**Affiliations:** 1 Research Group for Reproductive Medicine, IVF-Laboratory and Department of Gynecology, University of Cologne, Kerpener str. 34, 50931 Cologne, NRW Germany; 2 Department of Obstetrics and Gynecology, PLA General Hospital, Beijing, China; 3 Institute of Biology and Immunology of Reproduction, Bulgarian Academy of Sciences, Sofia, Bulgaria

**Keywords:** Cryopreservation, Breast cancer, Epithelial-mesenchymal transition, Metastasis, Angiogenesis, Chorioallantoic membrane

## Abstract

**Background:**

Ovarian tissue cryopreservation has a wide range of cancerous indications. Avoiding relapse becomes a specific concern that clinicians frequently encounter. The data about the comparative viability of cancer cells after cryopreservation are limited. This study aimed to evaluate the effect of cryopreservation on breast cancer cells.

**Methods:**

We used in-vitro cultured ZR-75-1 and MDA-MB-231 cell lines. Cell samples of each lineage were distributed into the non-intervened and cryopreserved groups. The cryopreservation procedures comprised programmed slow freezing followed by thawing at 100 °C, 60 s. Biological phenotypes and the related protein markers were compared between the two groups. The EVOS FL Auto 2 Cell Image System was used to monitor cell morphology. Cell proliferation, motility, and penetration were characterized by CCK-8, wound-healing, and transmembrane assay, respectively. The expression of Ki-67, P53, GATA3, E-cadherin, Vimentin, and F-Actin was captured by immunofluorescent staining and western blotting as the proxy measurements of the related properties. The chorioallantoic membrane (CAM) xenotransplantation was conducted to explore angiogenesis induced by cancer cells.

**Results:**

After 5 days in vitro culture, the cell concentration of cryopreserved and non-intervened groups was 15.7 × 10^4^ vs. 14.4 × 10^4^cells/ml, (ZR-75-1, *p* > 0.05), and 25.1 × 10^4^ vs. 26.6 × 10^4^ cells/ml (MDA-MB-231, *p* > 0.05). Some cryopreserved ZR-75-1 cells presented spindle shape with filopodia and lamellipodia and dissociated from the cell cluster after cryopreservation. Both cell lines demonstrated increased cell migrating capability and invasion after cryopreservation. The expression of Ki-67 and P53 did not differ between the cryopreserved and non-intervened groups. E-cadherin and GATA3 expression downregulated in the cryopreserved ZR-75-1 cells. Vimentin and F-actin exhibited an upregulated level in cryopreserved ZR-75-1 and MDA-MB-231 cells. The cryopreserved MDA-MB-231 cells induced significant angiogenesis around the grafts on CAM with the vascular density 0.313 ± 0.03 and 0.342 ± 0.04, compared with that of non-intervened cells of 0.238 ± 0.05 and 0.244 ± 0.03, *p* < 0.0001.

**Conclusions:**

Cryopreservation promotes breast cancer cells in terms of epithelial-mesenchymal transition and angiogenesis induction, thus increasing metastasis risk.

## Background

With the aim of fertility preservation, ovarian tissue cryopreservation (OTC) is currently the medical treatment of an increasing application [1]. The beneficiaries include the prepubertal, adolescent, and young adults diagnosed with malignant diseases e.g. gastrointestinal carcinoma, leukemia and breast cancer [1, 2]. Clinicians concern about the existence of disseminated cancer cells that are dormant in the ovaries before anti-cancer treatment [3]. However, data about effect of cryopreservation on viability of cancer cells are limited.

As reported, cryopreservation adversely affected the decidualization potential and cytokine production of human endometrial stromal cells [4]. The activity of xenobiotic metabolizing enzymes and responsiveness to enzyme-inducing agents reduced in cryopreserved human hepatocytes compared with that in freshly isolated cells [5]. However, cryopreserved umbilical cord blood mononuclear cells (UCB-MNCs) exhibit similar properties to those of fresh UCB in vitro and in vivo [6]. Endothelial progenitor cells derived from UCB-MNCs induced responses to cytokines and recovery of carotid artery injury analogous with those from peripheral blood of healthy volunteers [7].

Optimization of procedures of cryopreservation has an aim to improve the viability of post-thawing cells [8–10]. Concurrently, the vitality of veiled or dormant cancer cells should not be neglected. Concealed disseminated cancer cells are asymptomatic and are thought to be growth-arrested in G0 to G1 of cell cycle and thus in a quiescent state during the freezing process. These cells evade the immune response and are untreatable due to drug resistance [11].

This study aimed to evaluate the effect of cryopreservation on human breast cancer cells in the form of compacted fragments (as a model of solid tumors).

## Methods

### Cell lines and culture

Except where otherwise specified, all reagents were obtained from Sigma (Sigma Chemical Co., St. Louis, USA).

ZR-75-1 and MDA-MB-231 cell lines were purchased from American Type Culture Collection (Manassas, USA, ATCC® Numbers: CRL-1500™; HTB-26™, respectively). The cell lines were tested for mycoplasma contamination before being performed in this study using LookOut Mycoplasma PCR Detection Kit (Sigma-Aldrich, St. Louis, MO). Cells were in vitro cultured in AIM V Medium (Thermo Fisher Scientific, Waltham, USA) supplemented with 10% fetal bovine serum (FBS) and Amphotericin B at 37 °C in a humidified chamber with 5% CO_2_. Culture media were renewed every 48 h.

The process of using breast cancer cell monolayer to form the model tissue of a solid tumor was previously described [12]. Briefly, the in-vitro cultured cells after three times of cell passages were maintained in the culture medium for 10 days without cell passage. Culture medium was renewed every 24 h after a cell monolayer was formed. A cell scraper (Greiner Bio-one, Frickenhausen, Germany) was used to harvest and accumulate the cell layer as the model tissue for the followed cryopreservation. This method was also manipulated to collect the cancer cells for the chorioallantoic membrane (CAM) xenotransplantation and in vivo culture.

Cell samples of each lineage were distributed into the non-intervened and cryopreserved groups.

### Cryopreservation (freezing and thawing) of the model tissues

Cryopreservation of compacted fragments of cancer cells was implemented based on the protocols for cryopreservation of human ovarian tissue [13] with modifications and peculiarities as described below. The model tissues were frozen and thawed subjected to the process for ovarian strips.

The harvested tissues were kept for 5 min (ZR-75-1 cells) and 10 min (MDA-MB-231 cells) in the standard 5 ml cryo-vials (Thermo Fisher Scientific, Rochester, USA) previously filled by 4.5 ml freezing solutions (medium L-15 supplemented with 6% dimethyl sulfoxide, 6% ethylene glycol and 0.15 M sucrose) and precooled at 4 °C. Then the tissues were frozen using the IceCube 14S freezer (SyLab, Neupurkersdorf, Austria). The slow cooling profile started at − 6 °C with auto-seeding. The samples were then cooled from − 6 to − 34 °C at a rate of − 0.3 °C/min. At − 34 °C, the cryovials were plunged into liquid nitrogen and stored until thawing.

For the thawing of samples, the cryo-vial was removed from liquid nitrogen and held for 30 s at room temperature, then immersed in a 100 °C (boiling) water bath for 60 s. The exposure time in the boiling water was visually controlled by the presence of ice in the medium. Then the cryo-vial was removed from the boiling water when the ice was in the form of 1–2 mm apex, and the final temperature of the medium was between 4 and 10 °C. After 90% freezing medium was discarded within 10s, the cryo-vial was filled by 37 °C pre-warmed thawing solution (basal medium containing 0.5 M sucrose) and put into thermostat at 37 °C for 7 min and 15 min for ZR-75-1 and MDA-MB-231 cells, respectively, to remove the intracellular cryoprotectants. Then, approximately 90% thawing medium in the vial was expelled. The basal (culture) medium was slowly added into the vial holding the residual solution and the tissue inside, using the ‘dropping’ methodology for the stepwise rehydration [14]. The final concentration of sucrose was 0.05 M, resulting in an isotonic condition. After rehydration, the tissue fragments were digested by 6 ml 0.05% Trypsin-EDTA and maintained in the incubator for 5 min at 37 °C, 5% CO_2_. After washing and centrifugation, the cell pellet was resuspended in 10 ml culture medium by fully pipetting and then transferred into a 10 cm cell culture dish to allow adhesion overnight.

### Observation of cell proliferation and morphology

The non-intervened and cryopreserved group of cells were seeded at a concentration of 1 × 10^4^ cells/ml in 96-well plates and allowed to adhere overnight. Cell proliferation was measured using Cell Counting Kit-8 (CCK-8) and observed consecutively for 5 days. From day 1 to 5, ten μl CCK-8 solution was added to each well of one plate at a fixed time and incubated for 4 h, then the OD at 450 nm (reference 650 nm) was determined by a multimode reader machine (Tecan Group Ltd., Maennedorf, Zurich, Switzerland). Culture medium was renewed every 48 h. Results were plotted to draw a cell-growing curve with the time axis as the abscissa and the cell count as the vertical axis. Each experiment was repeated three times. For the morphology change, cells were maintained in the 10 cm culture dish to observe under microscopy each day. Images were taken by EVOS FL Auto 2 Cell Imaging System (Thermo Fisher Scientific).

### Assessment of cell motility and invasion

Cell migration and invasion were determined using the wound-healing and 3D transwell assay. The wound-healing assay was implemented with a well-established artificial gap on the confluent cell monolayer. A density of 1 × 10^6^ cells/ml in 140 μl suspension of both cell lines was seeded in a 35 mm μ-Dish ibidi Culture Insert (ibidi GmbH, Planegg, Bavaria, Germany) with 70 μl in each well, incubated for 24 h and obtained the cell layers. After removal of the insert, the μ-Dish was washed with PBS twice to remove cell debris and non-attached cells and filled with 2 ml of 1% FBS-supplemented cell-free medium. Time-lapse measurement of the wound area between the cell layers was conducted at time points 24, 48, and 72 h for ZR-75-1 and 2, 4, and 6 h for MDA-MB-231 cells to calculate cell front velocity. Experiments were carried out in triplicate at least three times.

Corning transwell inserts were used to accomplish the cell migration and invasion assay, according to our previous study [12]. Polycarbonate filters (6.5 mm in diameter, 8 μm pore size) were coated with type I rat tail collagen (100 μg/ml, BD Biosciences, Franklin Lakes, USA) for 1 h at 37 °C by the manufacturer’s protocol. The non-intervened and cryopreserved cells were resuspended and seeded into the upper compartment of the insert in the serum-free culture medium, respectively. ZR-75-1 cells were seeded at 2 × 10^5^ cells/well and cultured for 72 h; MDA-MB-231 cells were seeded 5 × 10^4^ cells/well and cultured for 8 h. The lower chamber was filled with 600 μl of the appropriate culture medium supplemented with FBS as a chemoattractant. After incubation, the upper insert with cells was washed with PBS, fixed with 4% formaldehyde, and permeabilized with methanol at room temperature. Cells were then stained with 0.1% crystal violet solution and were gently rinsed with PBS and wiped by cotton-tipped swabs then dried in the air. Penetrative cells went through the polymerized collagen layer to the bottom of the polycarbonate membranes and were counted in five different fields of view under a microscope. For the migration assay, cells were treated using the same procedure, except that the transwell membrane was not coated with collagen. Samples in each group ran in triplicate. Each experiment was performed at least three times.

### Immunofluorescent (IF) staining

Antibodies were purchased from Biolegend. Twenty-five× 10^4^ cells were first seeded on cover glasses in 6-well plates. After 48 h, the culture medium was aspirated, and cells were fixed with 2% paraformaldehyde for 20 min at room temperature. After washing twice by PBS, the cells were incubated in 0.5% Triton X-100 in PBS for 10 min for permeabilization and blocked by cell staining buffer (Biolegend, San Diego, USA) for 30 min. Then the coverslips were transferred into a humidified chamber and incubated with Alexa Fluor 488-conjugated anti-human Ki-67 antibody, Alexa Fluor 594 anti-human Epithelial cadherin (E-cadherin) antibody, Alexa Fluor 647 anti-GATA3 antibody and Alexa Fluor 488 anti-Vimentin antibody overnight at 4 °C, or with Alexa Fluor 488-conjugated Flash Phalloidin (F-Actin) in room temperature for 1 h. After washing twice, the coverslips were mounted on glass slides with 25 μl of mounting medium with 4′,6-diamidino-2-phenylindole (Abcam, Cambridge, UK). The slides were analyzed by a Leica SP8 confocal microscope. Images were taken using LAS X software (Leica Microsystems, Wetzlar, Germany).

### Western blotting (WB)

Cultured cells were incubated in Accutase at 37 °C for 5–10 min, followed by resuspension and centrifugation. Cell lysis was conducted using lysis buffer: RIPA buffer (Thermo Fisher Scientific) with protease inhibitor cocktail. Cell lysates were separated by centrifuging at 20000 g, 30 min at 4 °C. Protein concentrations were measured via Bradford test and adjusted to 20 μg/20 μl in one sample by 4X sodium dodecyl sulfate-containing laemmli sample buffer, then heated in boiling water for 5 min. Later, sodium dodecyl sulfate-polyacrylamide gel electrophoresis (SDS-PAGE) was applied to separate the total protein, and then separated protein was transferred on nitrocellulose membrane. We used the pre-cast 4–12% polyacrylamide gradient gels (Thermo Fisher Scientific) and the Trans-Blot® Turbo™ membrane (Biorad, Hercules, USA) in the transfer system according to the manufacturer instruction. After blocking, the membrane was incubated in primary antibodies diluted to 1:2000 by 5% Bovine Serum Albumin in PBST (0.1% Tween-20 in PBS) at 4 °C overnight. The P53, E-cadherin, GATA3, and Vimentin antibodies were purchased from Cell Signaling Technologies (Danvers, Massachusetts, USA). The following day, the fluorescent secondary antibodies (LI-COR, Lincoln, NE, USA) were used to incubate at room temperature for 2 h. Bands were visualized using Odyssey Clx (LI-COR). Image J software (http://developer.imagej.net) was used to estimate the band density.

### CAM-xenotransplantation: induction of angiogenesis and tumor growth

Preparation of the chick embryo chorioallantoic membrane (CAM) for transplantation of cancer cells were performed as described early [15, 16]. Briefly, fertilized eggs of White Leghorn chickens were purchased at a local hatchery and incubated at 37 °C–38 °C with 60% relative humidity for 3 days. On day 5, each egg was washed with warm 70% ethanol and opened a small window with 1.0 cm diameter on the sharp pole of the shell. We sealed the window by a 2 × 2 cm medical fabric tape only on the edge of the opening, and the egg was allowed to continue the incubation. The following day, a 1-mm-thick sterile silicone ring with an inner diameter of 5 mm was laid on the exposed chorioallantoic membrane. We divided 54 well-incubated 6-day-old chicken embryos randomly into four groups, 12 eggs in each group, and six as blank controls. Both the non-intervened and cryopreserved MDA-MB-231 model tissues were adjusted to two concentrations: 4 × 10^6^ and 8 × 10^6^ cells/egg. Then the four groups of samples were grafted into pre-treated chicken embryos on the relative avascular region of CAM: group 1: 4 × 10^6^ non-intervened cells; group 2: 8 × 10^6^ non-intervened cells; group 3: 4 × 10^6^ cryopreserved cells; group 4: 8 × 10^6^ cryopreserved cells. The blank control grafted 40 μl PBS. The five-millimeter inner diameter silicon rings were used to restrict the displacement of the grafts along with the chick embryo movement. The medical tape closed the window and continued to incubate for 6 days. The survival of the embryos, the tumor formation rate and the induction of angiogenesis were observed. The tumor with a diameter of ≥0.3 cm was considered positive, and the tumor formation rate was calculated. At the same time, the CAM xenograft specimens were fixed in situ with 4% paraformaldehyde and removed. The neovascularization in the tumor area was observed under a microscope on the 6th day of in vivo culture. The calculated field of blood vessels was set as the radial distribution within a radius of 1 cm from the grafted tissue. Image J software was applied to measure the area of vessels and CAM. The relative density of blood vessels was calculated by the formula: Vascular density = vasculature area/CAM area. Tumor volume was measured under an inverted microscope by the formula: Tumor volume = 1/2 × (major axis × minor axis^2^).

### Statistical analysis

Data analysis was executed with SPSS 23.0 software (IBM Corp., Armonk, USA). The student’s t-test was conducted to measure the differences between the cryopreserved and non-intervened groups. All statistical tests were 2-sided. Data are expressed as mean ± standard deviation (SD). The level of statistical significance was set at *p* < 0.05. The *p*-values < 0.05, < 0.01, < 0.001, and < 0.0001 were represented by one, two, three, and four asterisks on the bars in the figures, respectively. At multiple time points, the group effects were tested using generalized linear mixed models to investigate the dynamic effects of cryopreservation on cell migration (wound healing assay).

## Results

### Cell proliferation is invariable after cryopreservation

After 5 days of in vitro culture, the ZR-75-1 cell concentration of the cryopreserved and non-intervened groups was 15.7 × 10^4^ cells/ml and 14.4 × 10^4^ cells/ml, respectively, *p* > 0.05. The MDA-MB-231 cell concentration of the cryopreserved and non-intervened groups was 25.1 × 10^4^ cells/ml and 26.6 × 10^4^ cells/ml, respectively, *p* > 0.05, respectively, showing no statistical significance.

### ZR-75-1 cells exhibit morphology change

**Fig. 1:**
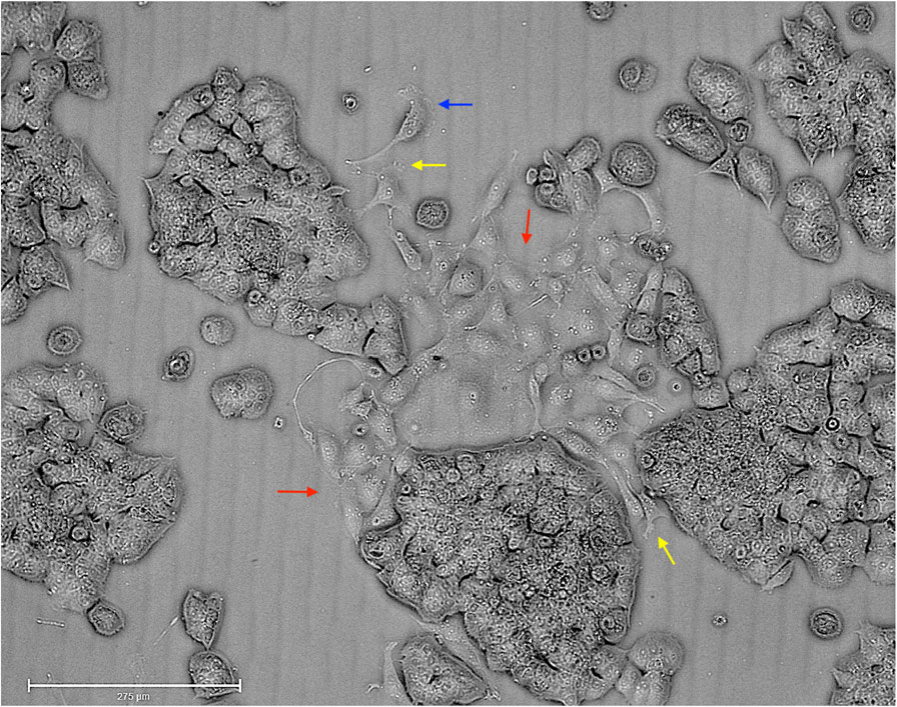
Morphological change of ZR-75-1 cells after cryopreservation. After cryopreservation, the cells were in vitro cultured in a 10 cm Petri dish. The majority of ZR-75-1 cells stayed typical grape-like clusters. The red arrows pointed out the cells transformed into a spindle shape and dissociated from the nearby cell clusters. The blue and yellow arrows pointed out the generated lamellipodia and filopodia, respectively. Magnification × 150

As shown in Fig. 1, a number of cryopreserved ZR-75-1 cells displayed morphology change from the typical grape-like cluster to fibroblast-like or spindle-shaped, and dissociated from the nearby cell cluster. The generation of filopodia and lamellipodia was observed. The compelling morphology changes are associated with the enhanced cell motility. Such cell characters were incapable to recognize in the cryopreserved MDA-MB-231 cells under the microscope due to its primitive morphology.

### Cryopreservation increases migrating capability and invasion of the cancer cells

Our data showed that the cancer cells after cryopreservation healed the wound area significantly more rapidly than before cryopreservation. For ZR-75-1 cells, the non-intervened group took over 72 h to close 100% of the gap, whereas the cryopreserved group healed the area within 72 h. For MDA-MB-231 cells, the non-intervened group closed 35% of the gap in 6 h, whereas the cryopreserved group covered 65% of the wound area, *p* < 0.05.

**Fig. 2:**
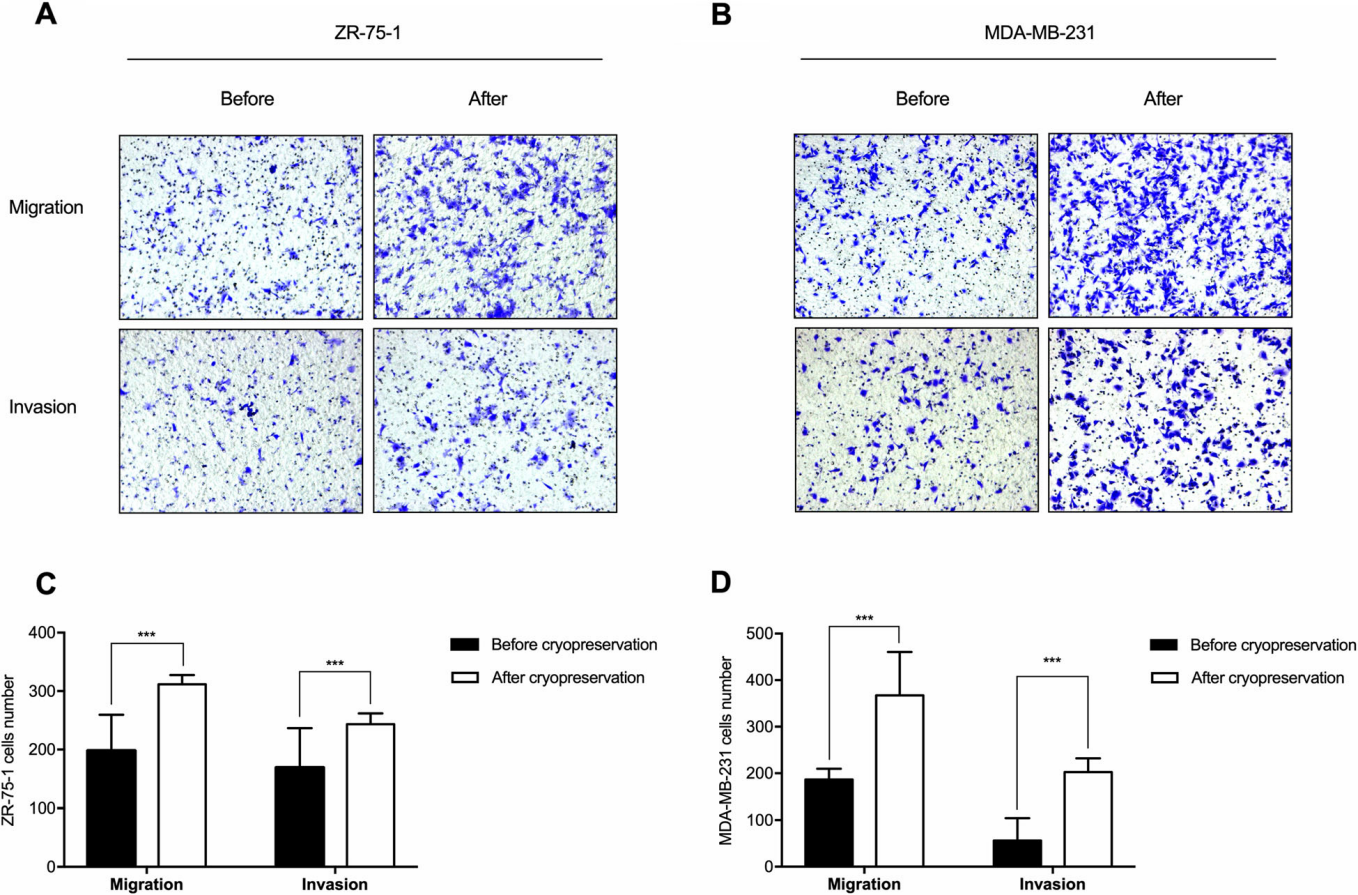
Increased transmembrane migration and invasion of cryopreserved breast cancer cells. **a** ZR-75-1 cells of the non-intervened group/before cryopreservation and the investigated group/after cryopreservation were seeded in transwell and cultured for 72 h. The number of stained cells on the bottom of the membrane after cryopreservation was significantly higher than before cryopreservation. **b** MDA-MB-231 cells of the group before and after cryopreservation were seeded in transwell and cultured for 8 h. The number of stained cells on the bottom of the membrane after cryopreservation was significantly higher than before cryopreservation. **c** and **d** Triplicate samples of each group from three independent experiments were included (*n* = 3). Data are analyzed using Student’s *t*-test, and expressed as mean ± SD. Magnification × 13.5. Significantly different at ^***^p < 0.001

By transwell assay, images of the stained cells on the bottom of the membrane were presented as photographic evidence of cell transmembrane migration and invasion. Data displayed that the cell dynamics and invasive capability were significantly enhanced in cancer cells after the cryopreservation treatment, as shown in Fig. 2. The number of migrated and invaded cells after 72 h (ZR-75-1) and 8 h (MDA-MB-231) culture was significantly higher in the cryopreserved group than the non-intervened group.

### Cryopreservation regulates the expression of protein Ki-67 and P53

Accordingly, the expression of multiple related proteins was the proxy assessment as evidence of the breast cancer cell phenotypes. By IF staining and WB, Ki-67 and P53 measurements were conducted in ZR-75-1 and MDA-MB-231 cells. The proportion of Ki-67 positive cells decreased after cryopreservation, showing 50.7% vs. 45.0%, *p* > 0.05, in ZR-75-1 cells, and 82.6% vs. 79.6%, *p* > 0.05, in MDA-MB-231 cells. However, the expression of P53 slightly increased after cryopreservation, exhibiting no statistical difference compared to before cryopreservation, *p* > 0.05.

### Cryopreservation induces loss of intercellular adhesion

**Fig. 3:**
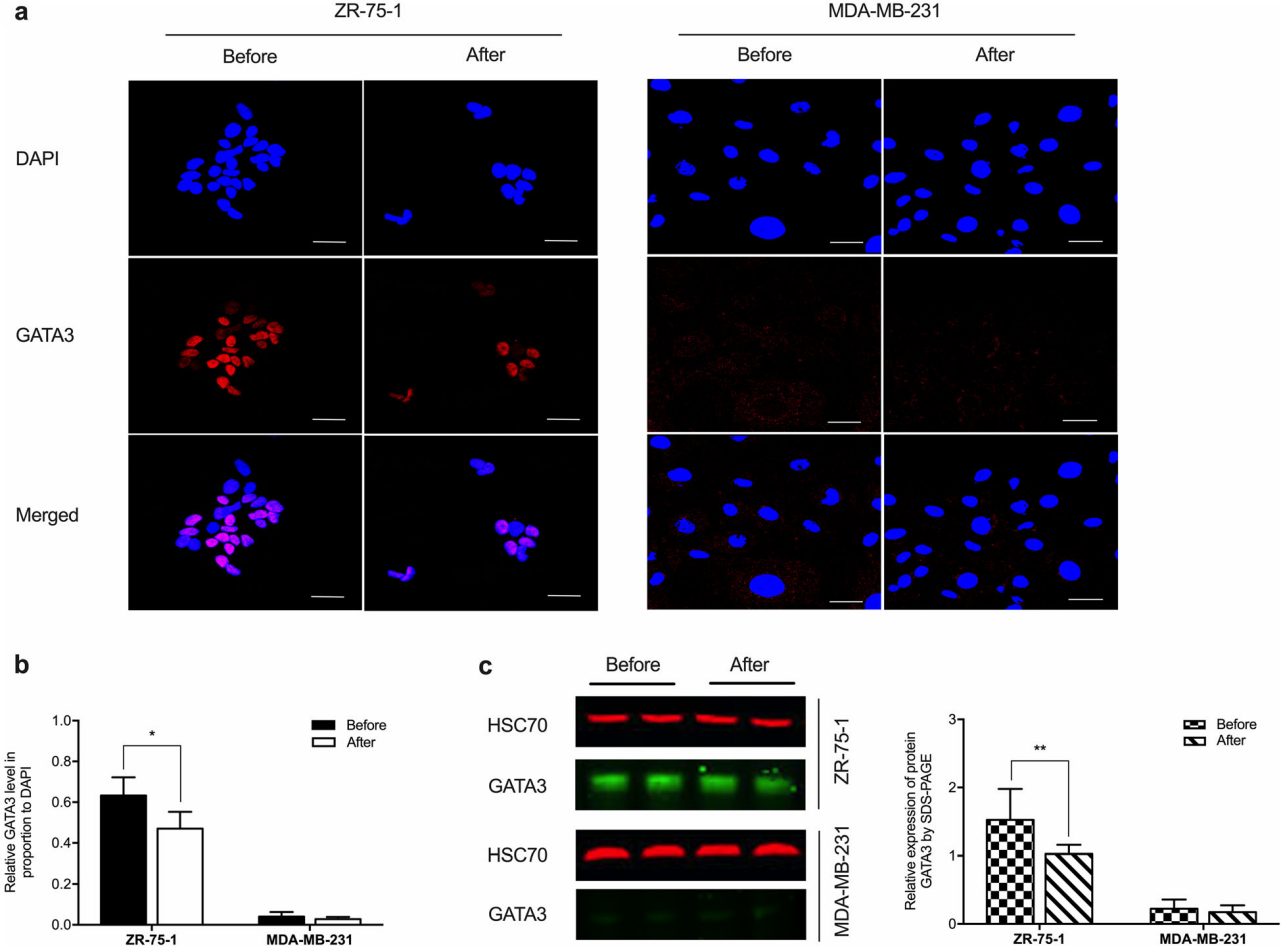
Expression of GATA3 in breast cancer cells before and after cryopreservation. **a** Cryopreserved and non-intervened ZR-75-1 and MDA-MB-231 cells were immunostained with the GATA3 marker. Cryopreservation led to diminished expression of GATA3 in ZR-75-1 cells. The confocal images showed that GATA3 expression in MDA-MB-231 cells was at an undetectable low level. Scale bar: 50 μm. **b** The intensity of the fluorescent signal in the cryopreserved cells significantly attenuated compared to the non-intervened samples. Data were analyzed by Student’s *t*-test and expressed with mean ± SD, ^*^p < 0.05. Triplicate samples were included in five independent experiments (*n* = 5). **c** Left panel: Cryopreservation downregulated GATA3 expression in ZR-75-1 cells. The blots of the protein expression in MDA-MB-231 cells were not capable of capturing. HSC70 was the loading control. Right panel: The protein expression level equaled the proportion of GATA3/HSC70. Data were calculated using Student’s t-test and expressed with mean ± SD from five independent experiments (n = 5). Significantly different at ^**^p < 0.01. The Odyssey Clx (LI-COR) was applied to visualize the bands. Image J software was used to measure the band intensity. These blots were cropped. Full-length blots/gels are presented in Supplementary Figure 1

The expression of GATA3 and E-cadherin was investigated, which involved in intercellular adhesion formation. GATA3 expression reduced significantly in ZR-75-1 cells after cryopreservation compared to before the treatment. The MDA-MB-231 cell line was of triple-negative molecular subtype; thus, the GATA3 expression was incapable of capturing (Fig. 3).

**Fig. 4:**
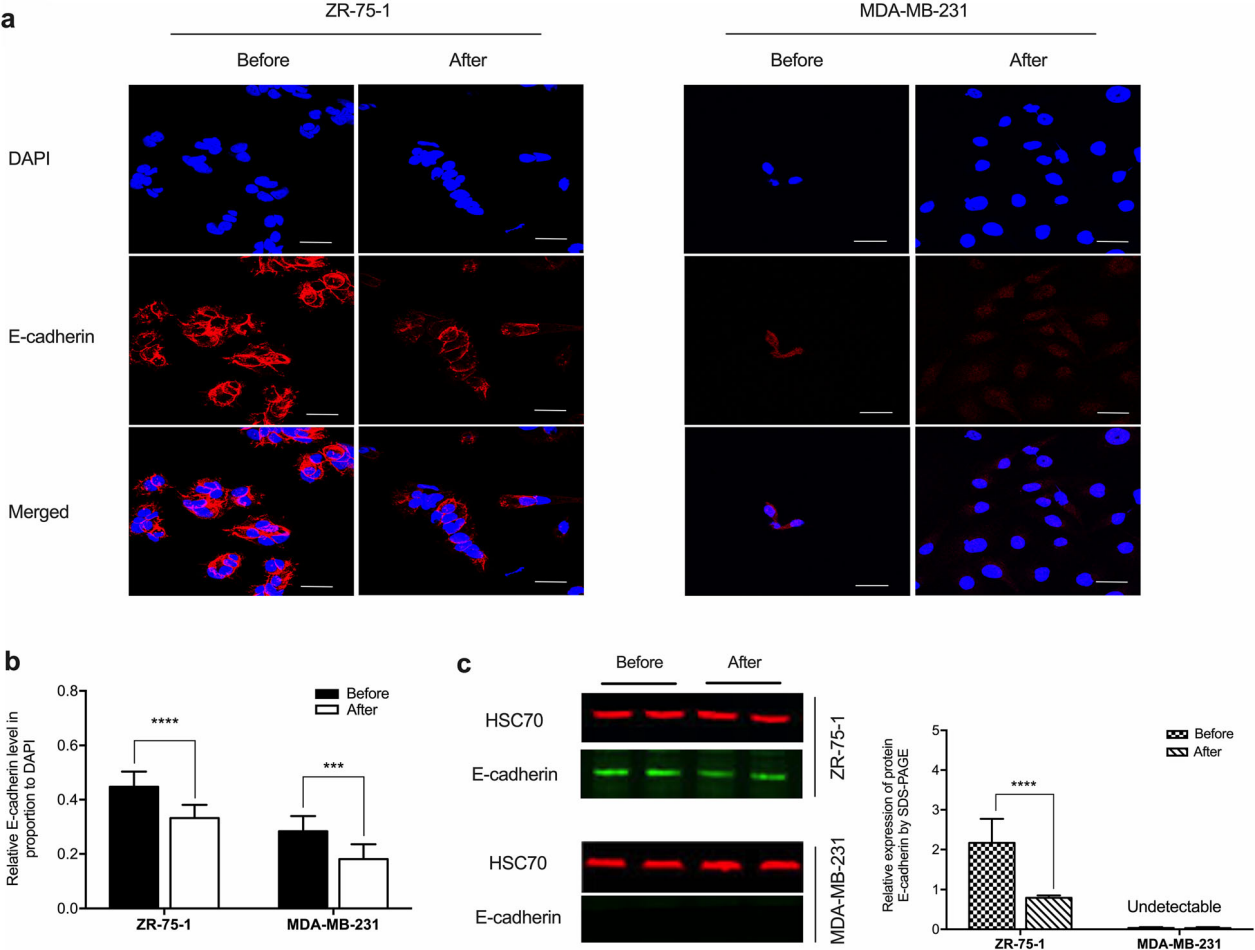
Expression of E-cadherin in breast cancer cells before and after cryopreservation. **a** Cryopreserved and non-intervened ZR-75-1 and MDA-MB-231 cells were immunostained with the E-cadherin marker. The expression of E-cadherin decreased in ZR-75-1 cells after cryopreservation. The confocal images showed that E-cadherin expression in MDA-MB-231 cells was significantly low and further attenuated after the cryopreservation treatment. Scale bar: 50 μm. **b** The intensity of the fluorescent signal in the cryopreserved cells significantly reduced compared to the non-intervened samples. Triplicate samples were included in five independent experiments (*n* = 5). Data were analyzed by Student’s *t*-test and expressed with mean ± SD. Significantly different at ^****^p < 0.0001 by ZR-75-1 cells, and ^***^p < 0.001 by MDA-MB-231 cells. **c** Left panel: Cryopreservation downregulated E-cadherin expression in ZR-75-1 cells. The blots of the protein expression in MDA-MB-231 cells were undetectable. HSC70 was the loading control. Right panel: The protein expression level equaled E-cadherin/HSC70 ratio. Data were calculated using Student’s *t*-test and expressed with mean ± SD from five independent experiments (*n* = 5). Significantly different at ^****^p < 0.0001. The Odyssey Clx (LI-COR) was applied to visualize the bands. Image J software was used to measure the band intensity. These blots were cropped. Full-length blots/gels are presented in Supplementary Figure 2

E-cadherin expression was affected by GATA3. The immunofluorescent signals significantly attenuated in the cryopreserved cells, representing the protein downregulation (Fig. 4). Our data indicated that cryopreservation led to the loss of intercellular adhesion in breast cancer cells.

### Cryopreservation enhances cell motility by upregulating Vimentin and F-actin

**Fig. 5:**
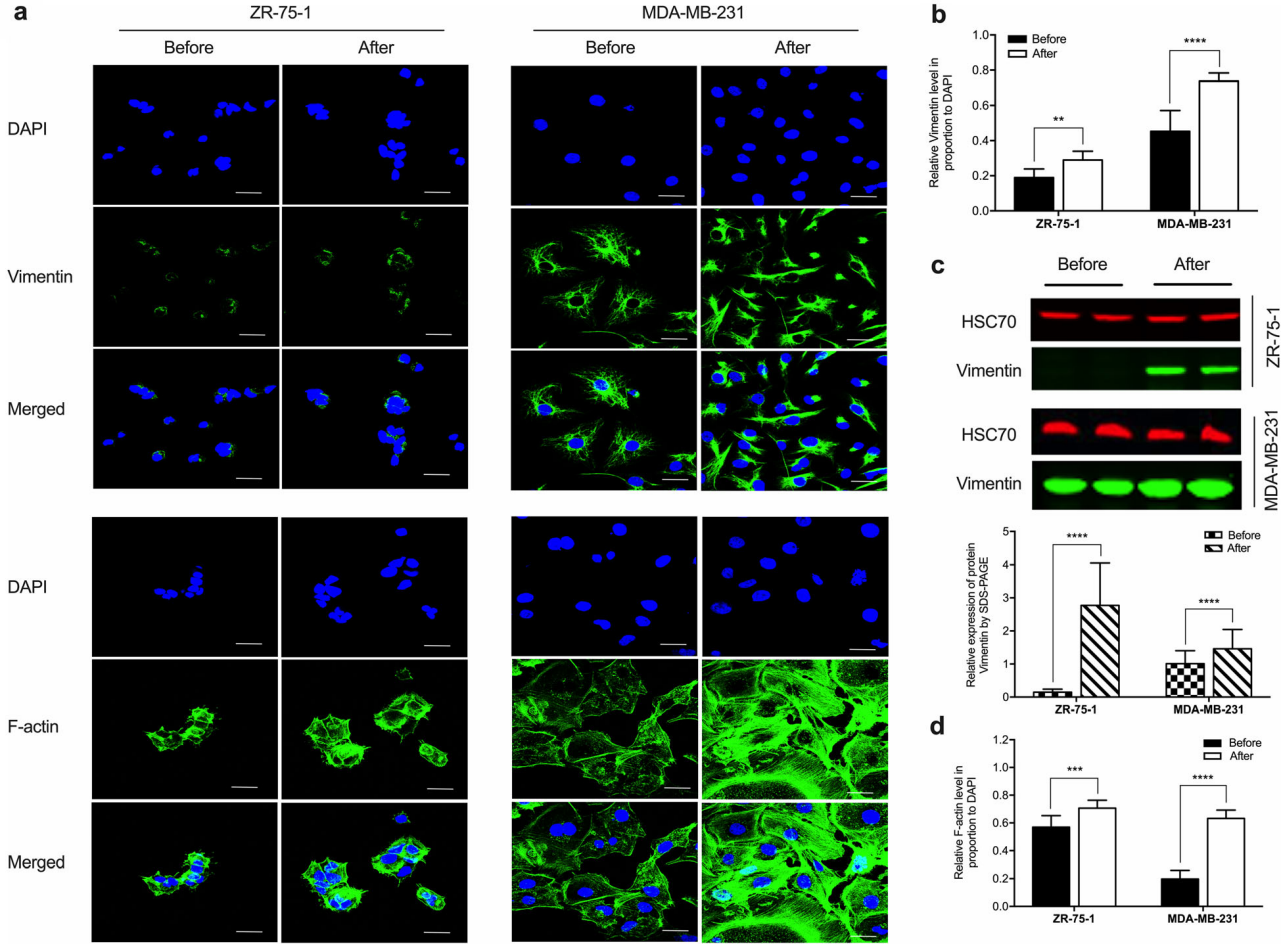
Expression of critical dynamic factors in breast cancer cells before and after cryopreservation. **a** Cryopreserved and non-intervened ZR-75-1 and MDA-MB-231 cells were immunostained with anti-Vimentin and anti- Phalloidin (F-actin) antibodies. The fluorescent images demonstrated that the protein expression was significantly upregulated in the cells after cryopreservation compared to before cryopreservation. Scale bar: 50 μm. **b** and **d** The histogram represented the relative expression of Vimentin and F-actin in the breast cancer cells, respectively. Triplicate samples were included in five independent experiments (*n* = 5). Data were analyzed by Student’s *t*-test and expressed with mean ± SD. ZR-75-1 before vs. after ^**^p < 0.01 for Vimentin, and ^***^p < 0.001 for F-actin. MDA-MB-231 cells before vs. after ^****^p < 0.0001 for Vimentin and F-actin. **c** Cryopreserved and non-intervened breast cancer cells were lysed and subjected to Western blotting with anti-Vimentin antibody (HSC70 as the loading control). Upper panel: Cryopreservation enhanced Vimentin expression, whereas the blot of the protein expression was undetectable in the non-intervened ZR-75-1 cells. Lower panel: The protein expression level equaled Vimentin/HSC70 ratio. Data were calculated by Student’s *t*-test and expressed with mean ± SD from five independent experiments (n = 5). Significantly different at ^****^p < 0.0001. The Odyssey Clx (LI-COR) was applied to visualize the bands. Image J software was used to measure the band intensity. These blots were cropped. Full-length blots/gels are presented in Supplementary Figure 3

The IF images demonstrated that Vimentin and F-actin expression significantly upregulated in the cells after cryopreservation compared to those before cryopreservation. By WB, Vimentin expression was undetectable in the non-intervened ZR-75-1 cells, whereas it was captured high in the cryopreserved cells. The protein level in MDA-MB-231 cells further increased after cryopreservation compared to before the treatment (Fig. 5), suggesting enhanced cell dynamics.

### Cryopreservationn stimulates angiogenesis and tumor growth

The survival rate of chick embryos inoculated by the non-intervened cells was > 90%, and that of the two groups inoculated by cryopreserved cells was > 80%, *p* > 0.05. The tumor formation rate was > 90% for the non-intervened and cryopreserved cancer cells, *p* > 0.05.

**Fig. 6:**
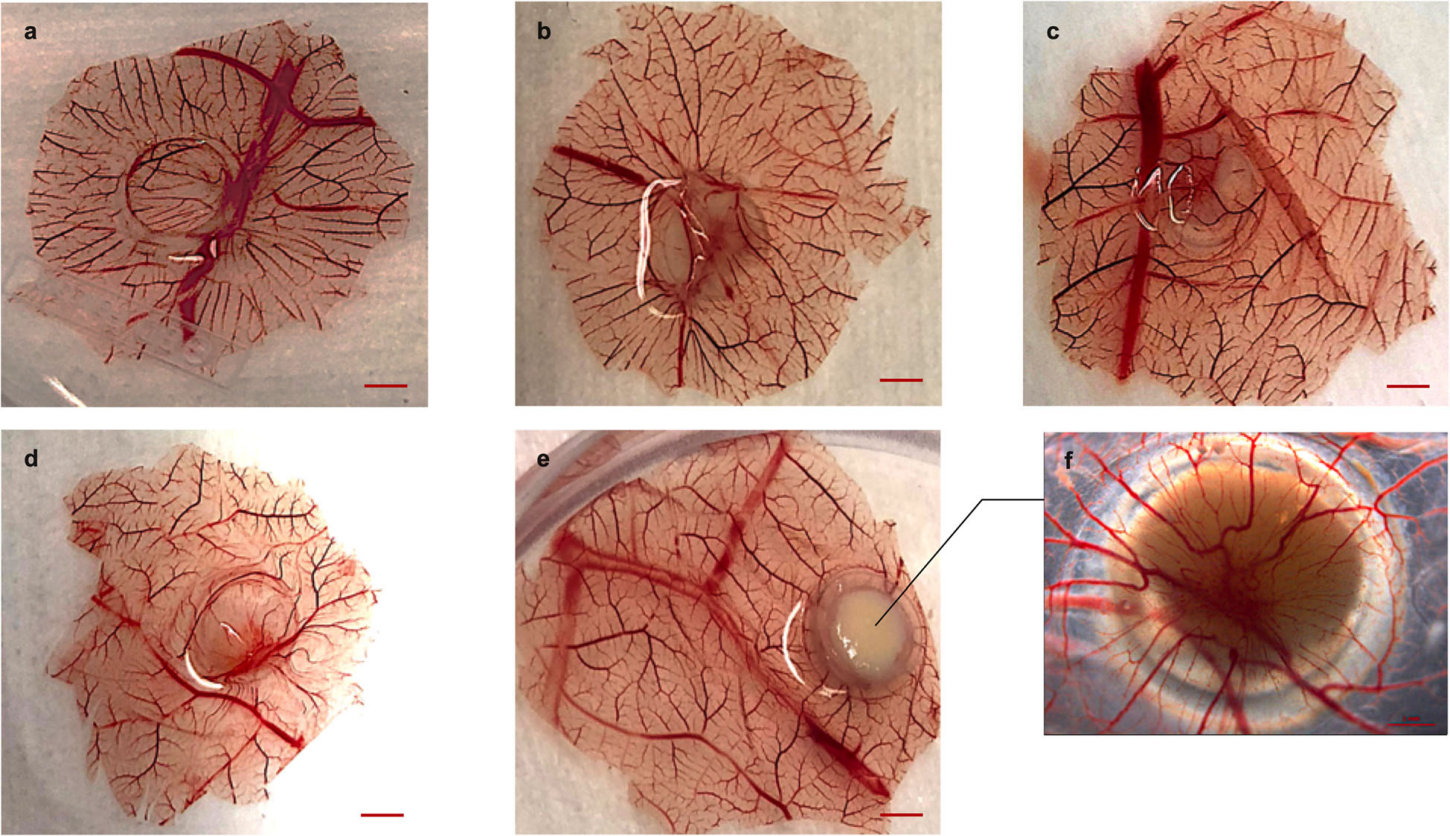
Angiogenesis on the CAM induced by MDA-MB-231 cells. The non-intervened cancer cells and the cells after cryopreservation were accumulated and transplanted on the relative avascular region of the CAM. **a** Negative control: 40 μl 1X Phosphate-Buffered Saline. **b** Representative sample of group 1: 4 × 10^6^ non-intervened cells. **c** Representative sample of group 2: 8 × 10^6^ non-intervened cells. **d** Representative sample of group 3: 4 × 10^6^ cryopreserved cells. **e** Representative sample of group 4: 8 × 10^6^ cryopreserved cells; Scale bar: 2.5 mm. **f** The reverse side of sample in (**e**). It was detected numerous radically distributed vasculature and vertically growth into the tissues, which was indicated by the reddishness at the middle of the transplanted sites. Scale bar: 1 mm. Image J software was applied to measure the area of vessels and CAM

In the blank samples, the disparity of the blood vessel morphology was not found between the inoculated and non-inoculated areas, presenting smooth and equably distributed. In groups 1, 2, 3, and 4, xenograft sites showed the radial distribution of blood vessels and an increased branching of the surrounding vasculature. Compared to the non-intervened group, it was observed in the cryopreserved groups a distinct growth of capillaries into the grafted tissue along with an increased number of peripheral blood vessels, which exhibited an intensive dendritic configuration (Fig. 6). The vascular area/ CAM area ratio of group 1, 2, 3, and 4 was 0.238 ± 0.05, 0.244 ± 0.03, 0.313 ± 0.03, and 0.342 ± 0.04, respectively. Thus, the vascular density of CAM transplanted by cryopreserved cells was higher, *p* < 0.0001. The variances of group 1 vs. group 2 and group 3 vs. group 4 were not statistically significant.

The tumor volume in groups 1, 2, 3, and 4 was 19.48 ± 3.07 mm^3^, 22.61 ± 6.99 mm^3^, 26.63 ± 6.44 mm^3^, and 46.48 ± 9.35 mm^3^, respectively. Tumor grafts in group 1 and group 2 were of small size, showing significant differences from those in group 3 and group 4, *p* < 0.05. The grafts in group 4 were of high volume compared to the other three groups, *p* < 0.0001, revealing that tumor growth was associated with the surrounded microenvironment and the autologous tumor burden.

## Discussion

Ovarian tissue cryopreservation and the following transplantation have served as a fertility preservation approach for over a decade. More and more cancer survivors access this treatment for fertility restoration [17, 18]. The effect of cryopreservation on cell viability and genetic regulation has been thoroughly investigated on various cell types [19], while the impact on cancer cells is largely unknown. Our study is the first to characterize the phenotypes and molecular changes of breast cancer cell lines undergoing cryopreservation.

Here, we tested ZR-75-1 cells of luminal A and aggressive MDA-MB-231 cells of triple-negative molecular subtype. To prevent intracellular crystallization during the process of cryopreservation, we used permeable cryoprotectants to protect the cells. The main cryoprotectants are high molecular alcohols: glycerol, ethylene glycol, propylene glycol, and dimethyl sulfoxide (DMSO). The ‘protective’ component is usually 10 to 12% of the total solution and is either a single ingredient (DMSO) or a mixture of DMSO and the other one of the glycols [20]. In our protocol, we used a mixture of two cryoprotectants, which we used to protect ovarian fragments included at least five types of cells. Our data showed that the protective effect of 12% DMSO was lower than that of a 12% multi-cryoprotectant solution (V. Isachenko, not published data). In this study, we further proved that cryopreservation using multi-cryoprotectants did not suppress cell growing ability reflected in the expression of Ki-67 and P53 in the cryopreserved and non-intervened breast cancer cells.

Epithelial-to-mesenchymal transition (EMT) is a reversible process, during which epithelial cells lose intercellular adherence and gain migratory and invasive properties to transdifferentiate to mesenchymal cells. We observed the decreased expression of GATA3 and E-cadherin in the cryopreserved cells. GATA3 functions as a critical transcriptional activator of E-cadherin to impede the phenotype transition between epithelial and mesenchymal cells, and suppresses metastasis and alters the tumor microenvironment in breast cancer [21]. E-cadherin is responsible for cell-cell adhesion. Wild-type E-cadherin downregulation is related to the reduction of intercellular adhesion [22]. Loss of E-cadherin is considered to be an elemental event in the process of EMT, which played a vital role in cancer metastasis [23]. It was reported that the expression of E-cadherin was suppressed in GATA3-knockout MDA-MB-231 cells [24]. Our data illustrated that the expression level of E-cadherin in ZR-75-1 cells was correlated to that of GATA3.

Vimentin and actin form the intermediate filament and microfilament, respectively, and participate in cell motility. Vimentin is the major cytoskeletal component of mesenchymal cells. F-actin also engages in the maintenance of cell shape. Since the induction of cell motility is considered the second phase of EMT [25, 26], we evaluated these two cytoskeletal proteins to reveal the mechanism of the enhanced cell moving after cryopreservation. Our data indicated that cryopreservation induced the improved migrating capability and invasion in breast cancer cells by upregulating the expression of Vimentin and F-actin and reorganizing intermediate filaments and microfilaments.

Angiogenesis is a vital process for tumor growth and spread. Our results revealed that cryopreserved breast cancer cells stimulated the generation of neovasculature. Subsequently, the cryopreserved grafts were of large volume after acquiring the newly established blood supply.

There are adverse effects observed at somatic cells cryopreservation: hypoxia is one of the most substantial effects besides intracellular Ca^2+^ concentration, osmotic disruption of cellular membranes, generation of reactive oxygen species, and lipid peroxidation [27]. Cryopreserved cancer cells experience an imbalance between oxygen delivery and consumption through the procedures of freezing and thawing. The condition of low oxygen tension activates the hypoxia-inducible factors (HIFs), increases the permeability of the mitochondrial membrane, causes mitochondrial swelling [28, 29], and enhances malignant phenotypes of cancer cells, that are positively correlated to cancer metastasis [30]. The transcription factors HIFs mediate the primary responses to hypoxia [31, 32]. Thereby, we inferred that cryopreservation altered GATA3 and E-cadherin expression through the activation of HIFs. HIFs also induce proteinases involved in the degradation of the extracellular matrix to accelerate the invasion then affect cell motility corresponding to cell migration and invasion, which is the first step of metastasis cascade [31].

Cell migration is associated with the metabolism of cellular energy. By cryopreservation, HIFs activation and mitochondria swelling increase glycolysis and thus sustain cancer metastasis [33–35]. Calcium regulates focal adhesion turnover, cytoskeletal reorganization, and other tumor cell movement processes through contact with multiple downstream proteins [36]. Whether HIFs and mitochondria induce the upregulation of Vimentin and F-actin still needs further research.

Tumors induce neovascularization by secreting various growth factors and proteinases [37, 38], several of which are the downstream proteins induced by HIFs. Besides, cancer cells cease mitosis and survive in dormancy under the condition of low temperature. A stable microvasculature constitutes dormant niches of cancer cells [39]. Angiogenesis accelerates the growth of quiescent breast cancer cells [40].

## Conclusions

Cryopreservation promotes breast cancer cells in terms of epithelial-mesenchymal transition and angiogenesis induction, thus increasing metastasis risk.

## Supplementary information

**Additional file 1: Figure S1.** The uncropped full-length western blotting images of Fig. 3. **a** The original blots/gels of the ZR-75-1 cell line. **b** The original blots/gels of the MDA-MB-231 cell line. Each image included four proteins, i.e., P53, E-cadherin, GATA3, and Vimentin, with 53kd, 125kd, 48kd, and 53kd of the expected molecular weight, respectively. HSC70 was used as the loading control. The first column on the left was the standard protein ladder. The molecular weights were labeled aside. Measurement of each protein marker occupied four adjacent tracks, of which the two on the left and the two on the right represented the expression of the relevant protein in the cell samples before and after cryopreservation, respectively. The white frames highlighted the green blots of GATA3 and red blots of HSC70, as shown in Fig. 3. Bands were visualized using the Odyssey Clx (LI-COR).

**Additional file 2: Figure S2.** The uncropped full-length western blotting images of Fig. 4. **a** The original blots/gels of the ZR-75-1 cell line. **b** The original blots/gels of the MDA-MB-231 cell line. Each image included four proteins, i.e., P53, E-cadherin, GATA3, and Vimentin, with 53kd, 125kd, 48kd, and 53kd of the expected molecular weight, respectively. HSC70 was used as the loading control. The first column on the left was the standard protein ladder. The molecular weights were labeled aside. Measurement of each protein marker occupied four adjacent tracks, of which the two on the left and the two on the right represented the expression of the relevant protein in the cell samples before and after cryopreservation, respectively. The white frames highlighted the green blots of E-cadherin and red blots of HSC70, as shown in Fig. 4. Bands were visualized using the Odyssey Clx (LI-COR)

**Additional file 3: Figure S3** The uncropped full-length western blotting images of Fig. 5. **a** The original blots/gels of the ZR-75-1 cell line. **b** The original blots/gels of the MDA-MB-231 cell line. Each image included four proteins, i.e., P53, E-cadherin, GATA3, and Vimentin, with 53kd, 125kd, 48kd, and 53kd of the expected molecular weight, respectively. HSC70 was used as the loading control. The first column on the left was the standard protein ladder. The molecular weights were labeled aside. Measurement of each protein marker occupied four adjacent tracks, of which the two on the left and the two on the right represented the expression of the relevant protein in the cell samples before and after cryopreservation, respectively. The white frames highlighted the green blots of Vimentin and red blots of HSC70, as shown in Fig. 5. Bands were visualized using the Odyssey Clx (LI-COR).

## Data Availability

The datasets used and/or analyzed during the current study are available from the corresponding author on reasonable request.

## Abbreviations

OTC: Ovarian tissue cryopreservation
UCB-MNCs: Umbilical cord blood mononuclear cells
FBS: Fetal bovine serum
CCK-8: Cell counting kit-8
IF: Immunofluorescent
E-cadherin: Epithelial cadherin
WB: Western blotting
CAM: Chorioallantoic membrane
SDS-PAGE: Sodium dodecyl sulfate-polyacrylamide gel electrophoresis
SD: Standard deviation
DMSO: Dimethyl sulfoxide
EMT: Epithelial-to-mesenchymal transition
HIFs: Hypoxia-inducible factors
