# Supplementary figures and images for: Increasing of malignancy of breast cancer cells after cryopreservation: molecular detection and activation of angiogenesis after CAM-xenotransplantation
Source: BMC Cancer. 2020 Aug 12;20:753. doi: 10.1186/s12885-020-07227-z (PMC7425039; doi:10.1186/s12885-020-07227-z)

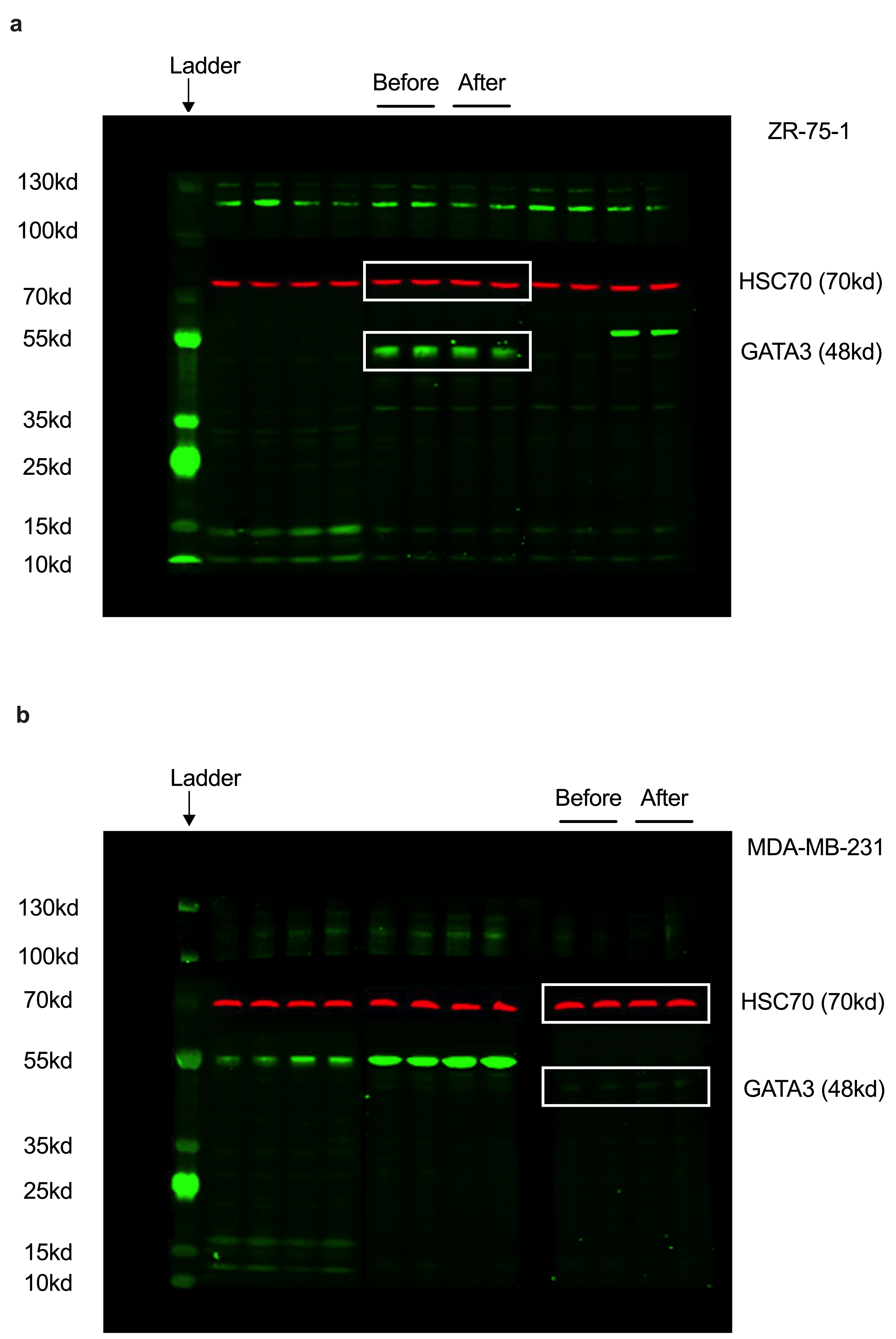

Supplement: Supplementary file 1 — Additional file 1: Figure S1. The uncropped full-length western blotting images of Fig. 3. a The original blots/gels of the ZR-75-1 cell line. b The original blots/gels of the MDA-MB-231 cell line. Each image included four proteins, i.e., P53, E-cadherin, GATA3, and Vimentin, with 53kd, 125kd, 48kd, and 53kd of the expected molecular weight, respectively. HSC70 was used as the loading control. The first column on the left was the standard protein ladder. The molecular weights were labeled aside. Measurement of each protein marker occupied four adjacent tracks, of which the two on the left and the two on the right represented the expression of the relevant protein in the cell samples before and after cryopreservation, respectively. The white frames highlighted the green blots of GATA3 and red blots of HSC70, as shown in Fig. 3. Bands were visualized using the Odyssey Clx (LI-COR). [file 12885_2020_7227_MOESM1_ESM.tiff]

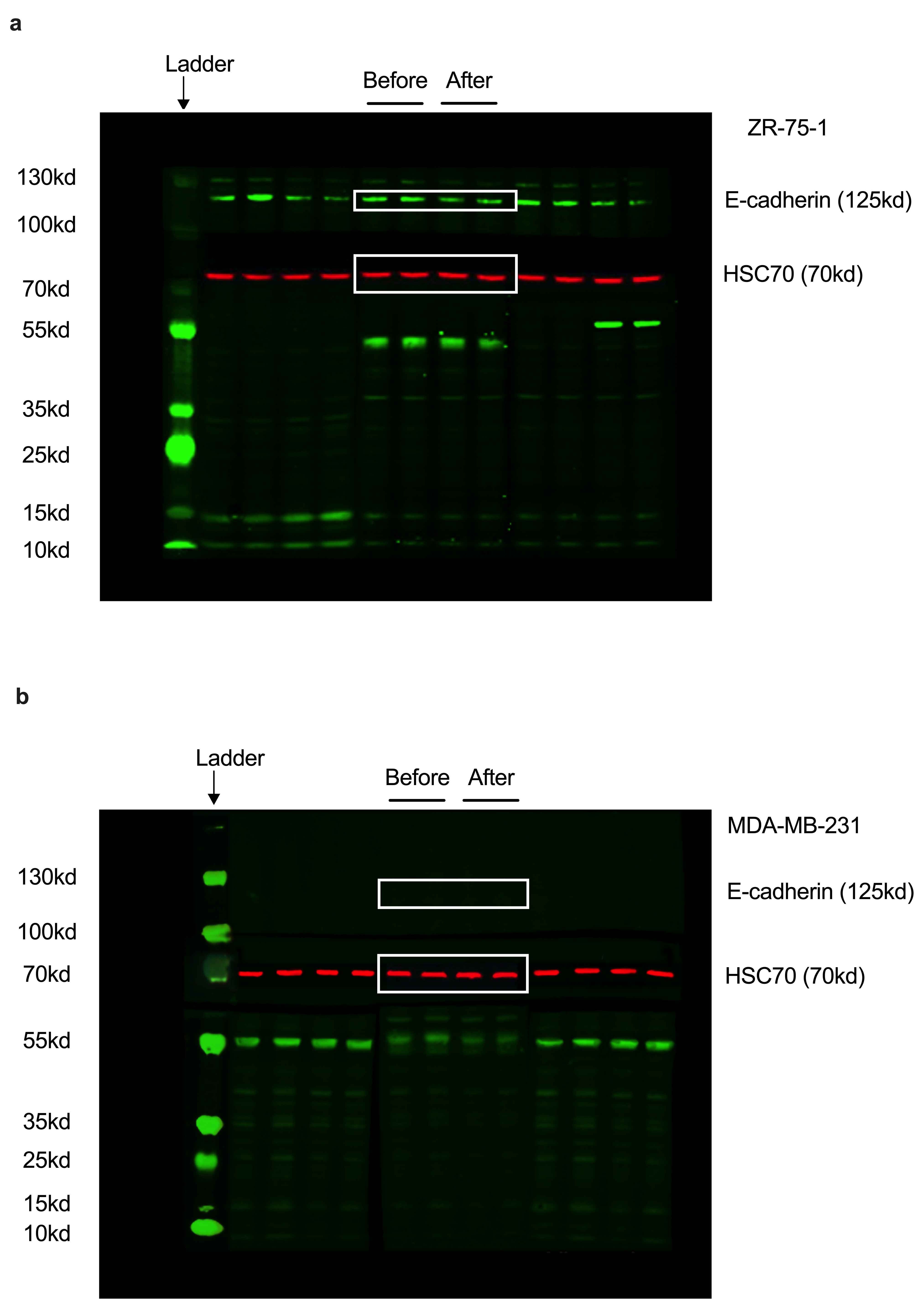

Supplement: Supplementary file 2 — Additional file 2: Figure S2. The uncropped full-length western blotting images of Fig. 4. a The original blots/gels of the ZR-75-1 cell line. b The original blots/gels of the MDA-MB-231 cell line. Each image included four proteins, i.e., P53, E-cadherin, GATA3, and Vimentin, with 53kd, 125kd, 48kd, and 53kd of the expected molecular weight, respectively. HSC70 was used as the loading control. The first column on the left was the standard protein ladder. The molecular weights were labeled aside. Measurement of each protein marker occupied four adjacent tracks, of which the two on the left and the two on the right represented the expression of the relevant protein in the cell samples before and after cryopreservation, respectively. The white frames highlighted the green blots of E-cadherin and red blots of HSC70, as shown in Fig. 4. Bands were visualized using the Odyssey Clx (LI-COR) [file 12885_2020_7227_MOESM2_ESM.tiff]

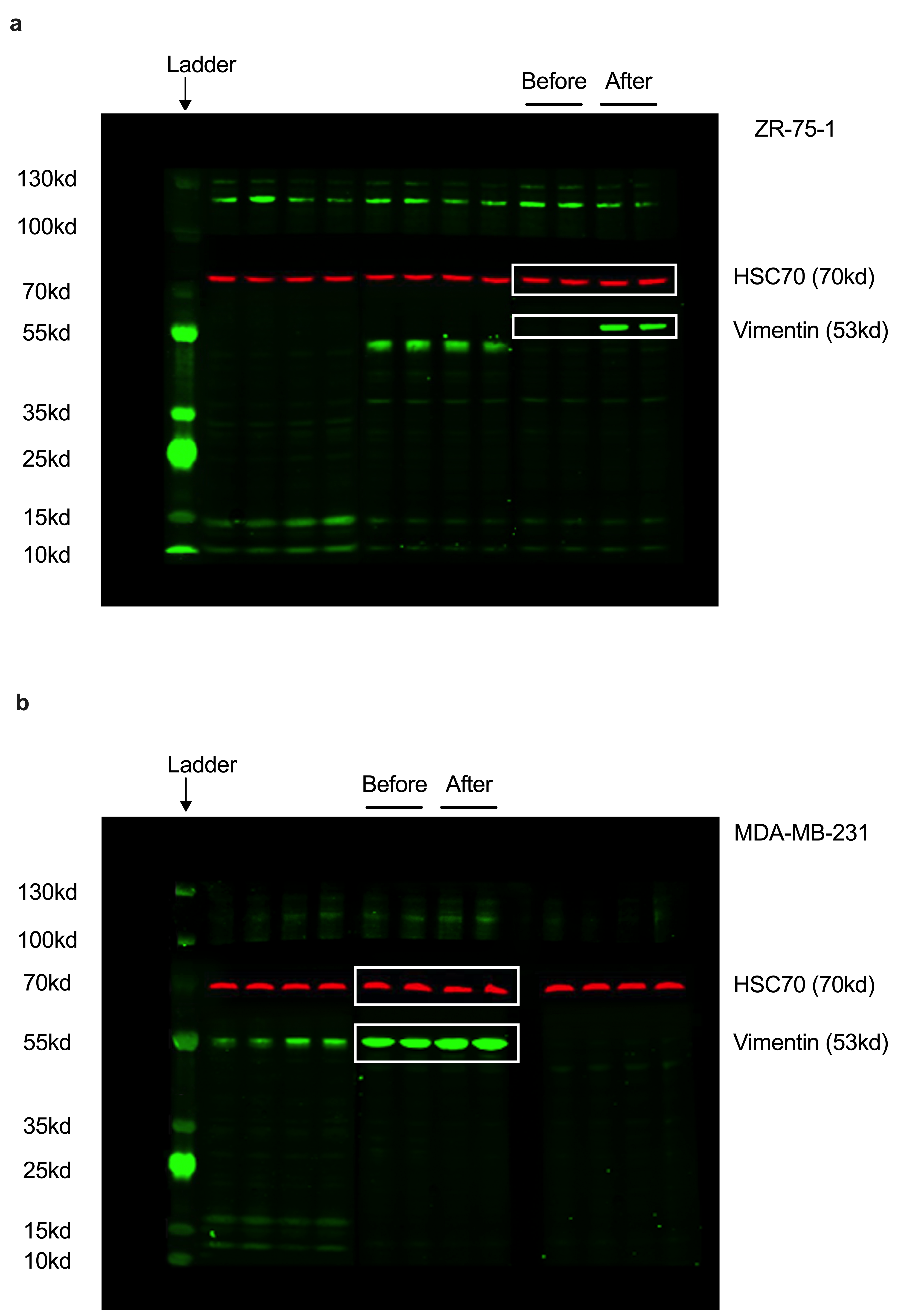

Supplement: Supplementary file 3 — Additional file 3: Figure S3 The uncropped full-length western blotting images of Fig. 5. a The original blots/gels of the ZR-75-1 cell line. b The original blots/gels of the MDA-MB-231 cell line. Each image included four proteins, i.e., P53, E-cadherin, GATA3, and Vimentin, with 53kd, 125kd, 48kd, and 53kd of the expected molecular weight, respectively. HSC70 was used as the loading control. The first column on the left was the standard protein ladder. The molecular weights were labeled aside. Measurement of each protein marker occupied four adjacent tracks, of which the two on the left and the two on the right represented the expression of the relevant protein in the cell samples before and after cryopreservation, respectively. The white frames highlighted the green blots of Vimentin and red blots of HSC70, as shown in Fig. 5. Bands were visualized using the Odyssey Clx (LI-COR). [file 12885_2020_7227_MOESM3_ESM.tiff]
